# Metagenomes of a Crystallizer Pond from Isla Cristina Saltern in Spain

**DOI:** 10.1128/mra.00039-23

**Published:** 2023-04-04

**Authors:** Alicia García-Roldán, Rafael R. de la Haba, Blanca Vera-Gargallo, Cristina Sánchez-Porro, Antonio Ventosa

**Affiliations:** a Department of Microbiology and Parasitology, Faculty of Pharmacy, University of Sevilla, Sevilla, Spain; Montana State University

## Abstract

The metagenomic sequences of the prokaryotic microbiota from the brine of a crystallizer pond with 42% (wt/vol) salinity of a saltern located in Isla Cristina, Huelva, southwest Spain, were obtained by Illumina. Haloarchaea and members of the bacterial genus *Salinibacter* were the most abundant prokaryotes.

## ANNOUNCEMENT

Solar salterns are multipond systems used for the commercial production of salt by evaporation of salted water, and they are excellent models for the study of the microbial communities living in a wide range of salt concentrations, from seawater to salt saturation (1). One of the salterns that has been extensively studied is Santa Pola saltern, located on the Mediterranean coast in Spain (2). In contrast, Atlantic salterns have received less attention. Isla Cristina saltern has been revealed as a source of interesting new prokaryotic microorganisms (3–12). In 2014, we reported early studies based on a metagenome obtained by pyrosequencing (Roche 454 GS-FLX technology) from the brine of an intermediate pond with 21% (wt/vol) salts (13). The analysis of this metagenome (designated IC21) indicated that *Euryarchaeota* (84%), mainly represented by the haloarchaeal genera *Halorubrum* (65.8%), *Natronomonas* (3.2%), and *Haloquadratum* (1.9%), was the most abundant phylum, followed by *Bacteroidota* (8.0%), dominated by the genus *Psychroflexus* (4.6%), and *Gammaproteobacteria* (7.0%), with the genus *Spiribacter* (1.1%) being the most abundant (14). In this report, we describe the sequencing of two metagenomic databases from the prokaryotic fraction of an Isla Cristina saltern crystallizer pond, in which salts precipitate, with a salinity of 42% (wt/vol). These were designated 20IC42-1 and 20IC42-2 and were obtained by Illumina technology.

Specifically, an 8-L brine sample was collected from the surface of a crystallizer pond with 42% (wt/vol) total salts (GPS coordinates 37°12′39.9′′N, 7°19′38.6′′W), on 20 July 2020. The pH and temperature of the brine at the time of sampling were 6.7 and 41.3°C, respectively. The sample was stored cold until transported to the laboratory and immediately was sequentially filtered through 5.0- and 0.22-μm-pore polycarbonate filters (Millipore, USA), obtaining four 0.22-μm filters that retained the desired prokaryotic fraction.

The total prokaryotic DNA was obtained with phenol-chloroform-isoamyl alcohol as previously described (15, 16). Two different DNA extractions were carried out from two 0.22-μm-pore filters each. Sequence libraries were constructed from the purified prokaryotic DNA using the Novogene NGS DNA library prep set (catalog no. PT004). Metagenomic sequencing was accomplished at a read length of 2 × 150 bp by Novogene Europe (Cambridge, United Kingdom) on an Illumina NovaSeq 6000 platform. Totals of 72,606,726 and 49,798,159 reads were obtained for the two replicates 20IC42-1 and 20IC42-2, respectively. Read quality control was performed with the “read_qc” module as implemented in MetaWRAP v.1.3.2 using default parameters (17). The taxonomic distribution was estimated from quality filter reads using Braken v.2.8 (18). The most abundant taxa were *Halorubrum* (34% and 32%), *Haloquadratum* (10% and 13%), and *Salinibacter* (10% and 8%) for the 20IC42-1 and 20IC42-2 metagenomic data sets, respectively.

These obtained metagenomic data sets will be used to describe the phylogenetic and functional diversity of a crystallizer pond of Isla Cristina saltern and to evaluate the biochemical pathways encoded under extremely-high-salinity conditions by haloarchaea and extremely halophilic bacteria.

### Data availability.

The sequences obtained in this project have been deposited in the NCBI Sequence Read Archive under accession no. SRR21894959 and SRR23092357 for 20IC42-1 and 20IC42-2, respectively.
